# Regional lymph node involvement and outcomes in appendiceal neuroendocrine tumors: a SEER database analysis

**DOI:** 10.18632/oncotarget.20362

**Published:** 2017-08-19

**Authors:** Amir Mehrvarz Sarshekeh, Shailesh Advani, Daniel M. Halperin, Claudius Conrad, Chan Shen, James C. Yao, Arvind Dasari

**Affiliations:** ^1^ Department of Gastrointestinal Medical Oncology, The University of Texas MD Anderson Cancer Center, Houston, TX 77030, USA; ^2^ Department of Surgical Oncology, The University of Texas MD Anderson Cancer Center, Houston, TX 77030, USA; ^3^ Department of Health Services Research, The University of Texas MD Anderson Cancer Center, Houston, TX 77030, USA

**Keywords:** appendix, neuroendocrine, right hemicolectomy, survival, tumor size

## Abstract

**Background:**

Appendiceal neuroendocrine neoplasms are most often diagnosed incidentally during appendectomy. The need for subsequent right hemicolectomy (RHC) is determined based on the risk of regional lymph node (LN) involvement. Tumor size has historically been used as an indicator of this risk, but controversy remains regarding its cut off. Furthermore, the impact of RHC on survival is unclear.

**Methods:**

We used the SEER database to identify patients diagnosed with appendiceal neuroendocrine tumors.

**Results:**

Of 1731 patients, 38.0% had well-differentiated neuroendocrine tumors (WDNETs), 60.8% had mixed histology tumors (MHTs), and 1.2% had poorly differentiated neuroendocrine carcinomas (PDNECs). In patients with WDNETs and MHTs who had adequate lymphadenectomy, higher rates of LN involvement were noted for tumors size 11–20 mm than ≤10 mm (56.8% vs. 11.6%, *p* <0.001; 32.9% vs. 10.4%, *p*=0.004, respectively). The type of surgery did not affect OS in cases with MHTs with LN involvement (HR 1.00; 95% CI, 0.53–1.89; *p* =0.99). Patients with regionally advanced WDNET showed excellent survival and only 3 patients (out of 118) died from cancer within 10 years.

**Conclusions:**

10 mm appears to be a more appropriate cutoff than 20 mm for predicting LN metastasis in appendiceal NETs. Cases with WDNETs and nodal involvement demonstrate overall excellent prognosis irrespective of type of surgery (i.e. RHC may not improve outcome). In MHTs with LN metastases, survival is markedly worse in spite of RHC. The role of adjuvant therapy should be evaluated in this subset.

## INTRODUCTION

Neuroendocrine neoplasms are the most common malignancies in the appendix. This heterogeneous group of tumors encompasses a wide spectrum of histologic types with different behaviors. The 2010 World Health Organization (WHO) Classification of Tumors of the Digestive System categorizes neuroendocrine neoplasms as well-differentiated neuroendocrine tumors (WDNET), poorly differentiated neuroendocrine carcinomas (PDNEC), and mixed adenoneuroendocrine carcinomas (MANEC) [1]. These subtypes are characterized by different morphological, clinical, and prognostic features. WDNETs usually have an indolent course and favorable long-term outcomes, although they can metastasize to lymph nodes and distant locations [2, 3]. MANEC is defined as a tumor with both neuroendocrine and epithelial components in which each component represents at least 30% of neoplastic tissue. MANECs, which also include goblet cell carcinoids, are often aggressive and have a prognosis comparable to that of adenocarcinoma of the colon [4–6]. Since the ratio of the histological components is not recorded in SEER database, we classified tumors with both neuroendocrine and epithelial components as “Mixed histology tumors (MHTs)”. PDNECs are exceedingly rare, have often metastasized at the time of presentation, and have a poor prognosis [5, 7, 8].

Appendiceal neuroendocrine neoplasms are usually discovered incidentally during appendectomy performed for other reasons, typically acute appendicitis [9–11]. In these cases, clinicians face a challenge when deciding whether to consider the simple appendectomy sufficient or to proceed with a second surgery, i.e., right hemicolectomy (RHC) with regional lymph node dissection. This decision is usually based on an assessment of the risk of regional lymph node involvement and/or metastatic disease. Although several pathologic features have been proposed to guide this decision, tumor size is the principal factor used to determine the need for RHC [2, 11, 12]. Historically, WDNET tumors larger than 20 mm have been thought to pose a significant risk of locoregional involvement and therefore to require treatment with RHC; smaller tumors were not expected to metastasize and were thought to be safely managed with simple appendectomy. This approach is based on small retrospective studies mainly evaluating “carcinoid” tumors of appendix [11, 13, 14]. The 20-mm cutoff has been widely accepted and used to formulate the treatment guidelines of the National Comprehensive Cancer Network, the European Neuroendocrine Tumor Society, and the North American Neuroendocrine Tumor Society [15–17]. These guidelines also recommend considering RHC in tumors larger than 10 mm when they have features that may predict highly aggressive behavior, such as lymphovascular invasion, positive surgical margins, invasion of the mesoappendix, and atypical/or mixed histology. However, some of these features may be inevaluable in appendectomy specimens. More importantly, it is unclear whether RHC is truly beneficial to patients with regionally advanced and node-positive disease, especially those with appendiceal WDNETs, who usually have an excellent prognosis.

We aimed to investigate the associations between the clinicopathologic features of appendiceal neuroendocrine tumors (including tumor size) and the risk of nodal metastases using the Surveillance, Epidemiology, and End Results (SEER) database. In this large population-based dataset, we also evaluated the associations of patient demographic, tumor characteristics and type of surgery (appendectomy versus RHC) with cancer-specific survival in each histologic subtype.

## RESULTS

**Figure 1:**
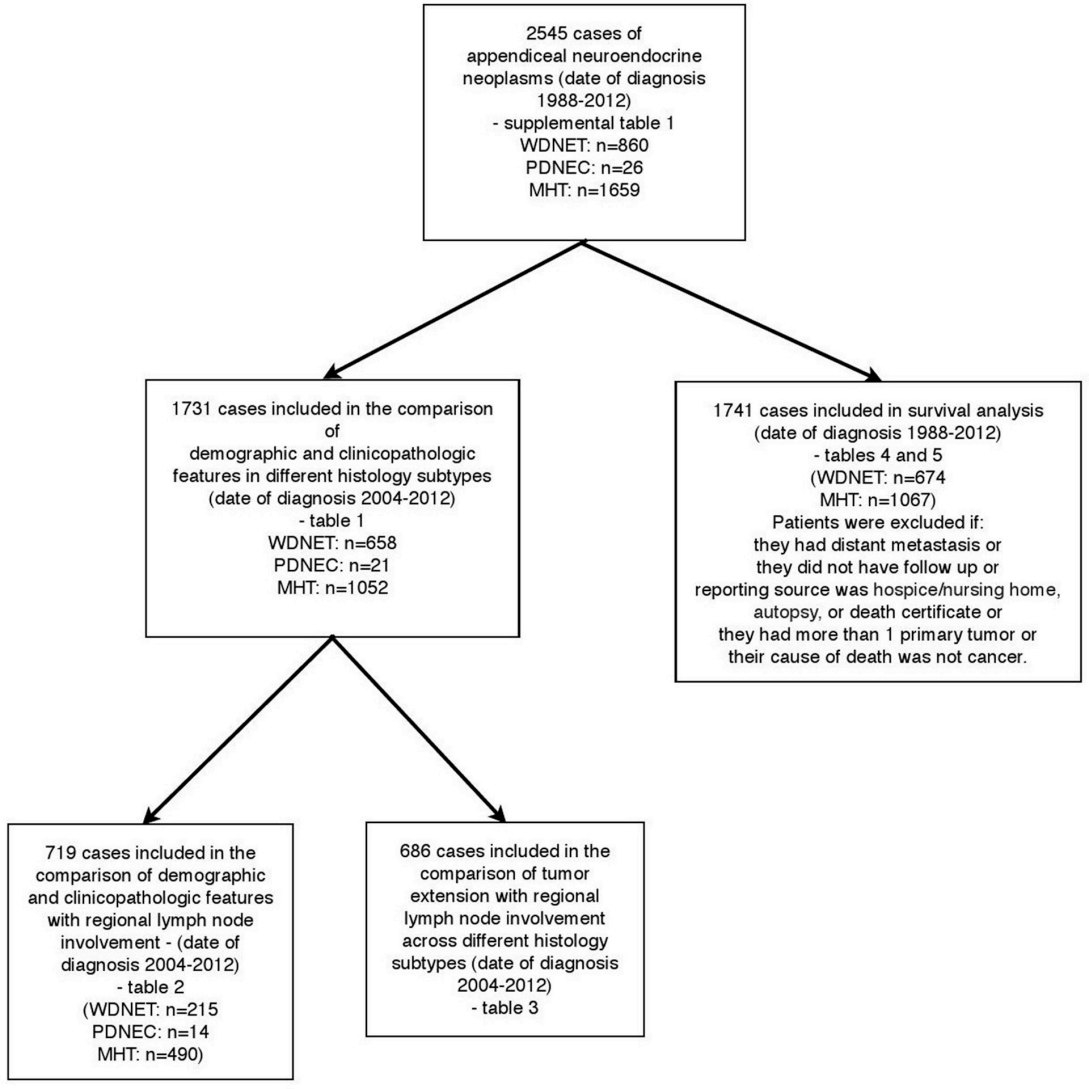
Patients included in our study WDNET, well-differentiated neuroendocrine tumor; PDNEC, poorly differentiated neuroendocrine carcinoma; MHT, mixed histology tumor.

A total of 2545 patients (860 with WDNET, 26 with PDNEC, and 1659 with MHT) who were diagnosed from 1988 to 2012 were initially selected for further analysis based on tumor histology (Supplementary Table 1). Using the inclusion and exclusion criteria described in the methods section, subsets of patients were chosen for each further analysis (Figure 1): 1731 patients diagnosed from 2004 to 2012 for the comparison of demographic and clinicopathologic features (Table 1); 719 patients diagnosed from 2004 to 2012 for the comparison of demographic and clinicopathologic features with regional lymph node involvement (Table 2); 686 patients for comparison of tumor extension and regional lymph node involvement (Table 3); and 1741 patients diagnosed from 1988 to 2012 for the survival analyses. Due to very small number of cases with PDNEC and per SEER Program Data Use-Agreement, we avoided reporting data regarding this histologic subtype in Tables 1, 2, and 3 to protect patient privacy.

**Table 1:** Demographic and clinicopathologic characteristics of 1731 patients with appendiceal neuroendocrine malignancies by histologic type (date of diagnosis 2004–2012)

Variable	No. of patients (%)				*P*
	WDNET		MHT^a^		
Median age at diagnosis		n=658		n=1052	**<0.001**
		42 y		56 y	
Sex		n=658		n=1052	
Female		418 (63.5)		516 (49.0)	**<0.001**
Male		240 (36.5)		536 (51.0)	
Race		n=642		n=1052	0.75
White		565 (88.0)		922 (87.6)	
Black		45 (7.0)		82 (7.8)	
Other^b^		32 (5.0)		48 (4.6)	
Tumor size^c^		n=571		n=660	**<0.001**
≤10 mm		296 (51.8)		177 (26.8)	
11–20 mm		164 (28.7)		178 (26.9)	
>20 mm		111 (19.4)		305 (46.2)	
T stage^c^		n=578		n=996	**<0.001**
T1	T1a	292 (50.5)		139 (14.0)	
	T1b	147 (25.4)			
T2		82 (14.2)		83 (8.3)	
T3		33 (5.7)		559 (56.1)	
T4		24 (4.2)	T4a	114 (11.4)	
			T4b	97 (9.7)	
			T4 NOS	4 (0.4)	
Nodal metastases		n=616		n=1005	0.21
No		490 (79.5)		825 (82.1)	
Yes		126 (20.5)		180 (17.9)	
Distant metastases		n=622		n=1015	**<0.001**
No		592 (95.1)		890 (87.7)	
Yes		30 (4.8)		125 (12.3)	

Abbreviations: WDNET: well-differentiated neuroendocrine tumor; MHT: mixed histology tumor;^a^ Includes goblet cell carcinoid.

^b^ Other: American Indian, Alaska Native, Asian/Pacific Islander, unknown.

^c^ Staging (TNM) and tumor size are based on clinical, imaging, and/or pathologic information.

**Table 2:** Association of demographic and clinicopathologic features with regional lymph node involvement * in patients who had adequate lymphadenectomy (date of diagnosis 2004–2012)

Characteristic	No. node-positive ^a^(%)	No. node-negative (%)	*P*
	(n=302)	(n=417)	
Median age at diagnosis	56 y	55 y	0.48
Sex	n=302	n=417	**0.01**
Female	174 (44.4)	218 (55.6)	
Male	128 (39.1)	199 (60.9)	
Race			0.45
White	271 (42.1)	372 (57.9)	
Black	22 (45.8)	26 (54.2)	
Histology	n=302	n=417	**<0.001**
WDNET	120 (55.8)	95 (44.2)	
MHT	170 (34.7)	320 (65.3)	
Tumor size^a^	n=235	n=288	**<0.001**
≤10 mm	10 (11)	81 (89)	
11–20 mm	69 (44)	88 (56)	
>20 mm	156 (56.7)	119 (43.3)	

* Patients were counted as node negative if they had adequate lymphadenectomy with 12 negative lymph node.

Abbreviations: WDNET: well-differentiated neuroendocrine tumor; MHT: mixed histology tumor

^a^ Nodal status and tumor size were based on pathologic data only.

**Table 3:** Proportion of patients who underwent adequate lymph node dissection * and had nodal metastases by tumor extension and tumor size (date of diagnosis 2004-2012)^a^

	WDNET			MHT
		No. LN-positive/No. patients examined (%)		No. LN-positive/No. patients examined (%)
T stage		n=199		n=483
T1	T1a	4/42 (9.5)		18.2 (8/44)
	T1b	36/63 (57.1)		
T2		37/51 (72.5)		18.5 (5/27)
T3		79.1 (19/24)		22.8% (64/280)
T4		78.9 (15/19)	T4a	51.5 (33/64)
			T4b	88.1 (59/67)
			T4 NOS	100.0 (1/1)
Tumor size		n=194		n=316
≤10 mm		11.6 (5/43)		10.4 (5/48)
11–20 mm		56.8 (42/74)		32.9 (27/82)
>20 mm		76.6 (59/77)		46.2 (86/186)

Abbreviations: WDNET: well-differentiated neuroendocrine tumor; MHT: mixed histology tumor; LN: lymph node; NOS: not otherwise specified.

^a^Tumor size and tumor extension data are based only on pathologic information.

* Patients were counted as node negative if they had adequate lymphadenectomy with 12 negative lymph node.

### Demographic and clinicopathologic features across different histologic subtypes

Of the 1731 patients diagnosed with appendiceal neuroendocrine neoplasms, 944 (54.5%) were female, and 1506 (87.0%) were white (Table 1). Their median age was 53 years, and the median tumor size was 15 mm. Patients with WDNETs tended to be younger (median age, 42 years), to be female (63.5%), and to have a lower T stage (90.0% had T1 or T2 tumors), smaller tumor size (51.8% had tumors ≤10 mm), and a lower rate of distant metastases than the other histologic types (all *p* <0.001). Overall, 319 (19.2%) of 1658 evaluable cases had clinical and/or pathologic evidence of nodal metastasis, and 166 (10.0%) had evidence of distant metastases.

### Associations between demographic and clinicopathologic features with regional lymph node involvement in each histologic subtype

Table 2 demonstrates the associations between demographic and clinicopathologic features with node positive and node negative disease. While age, sex, and race were not associated with regional node involvement, both histological subtype (55.8% node-positive in WDNETs vs. 85.7% in PDNECs and 34.7% in MHTs) and tumor size (11% node-positive in tumors ≤10 mm vs. 44% node-positive in tumors 11–20 mm and 56.7% in tumors >20 mm) were very strongly associated with nodal metastasis (*p* <0.001).

Next, we assessed the association of tumor size (and tumor extension, T stage) with regional lymph node involvement (Table 3) in each histological subtype. Nearly all PDNECs (19/20) were larger than 20 mm and displayed regional lymph node involvement. In both WDNETs and MHT, significantly higher rates of regional lymph node involvement were noted in 11-to-20-mm tumors than in smaller tumors (WDNETs, 56.8% vs. 11.6%, *p* <0.001; MHTs, 32.9% vs. 10.4%, *p* =0.004). In all histologic subtypes, as expected, the percentage of cases with regional lymph node involvement increased as T stage progressed since T stage correlates very closely with tumor size in appendiceal NETs.

### Associations between clinicopathologic characteristics, type of surgery and cancer specific survival

**Figure 2:**
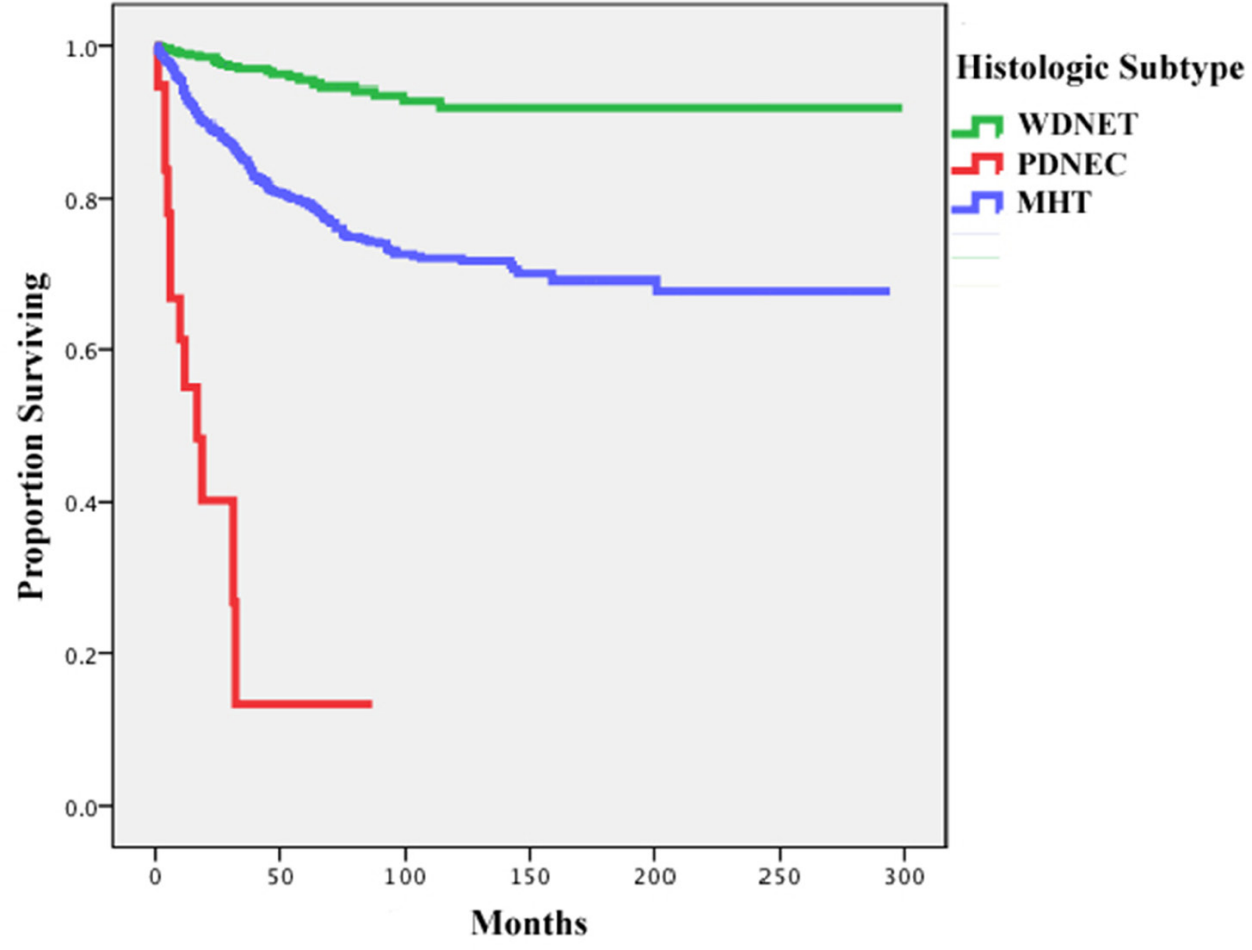
Cancer-specific survival rates of all included patients with appendiceal neuroendocrine malignancies by histologic subtype WDNET, well-differentiated neuroendocrine tumor; PDNEC, poorly differentiated neuroendocrine carcinoma; MHT, mixed histology tumor.

We included 1741 patients with localized or regionally advanced disease in survival analyses (Figure 1). The median follow-up time among these patients was 44 months. The cumulative cancer-specific survival rates according to histologic subtype are shown in Figure 2. Patients with WDNETs had significantly higher 10-year survival rates than did those with MHTs (92.63% (95 CI=88.25%-95.41% vs. 78.18%, 95% CI=74.69%-81.26%, *p* <0.001), but none of the patients with PDNEC survived for 10 years (Table 4).

**Table 4:** Comparison of 10-year cancer-specific survival rates of all patients with WDNET and MHT (date of diagnosis 1988–2012)

Histologic type	No. of patients	No. of deaths	10-year survival rate	*P*^*a*^
**WDNET**	674	54	92.63%	<0.001
**MHT**	1067	296	78.18%	

^a^ log-rank test.

Abbreviations: WDNET: well-differentiated neuroendocrine tumor; MHT: mixed histology tumor.

Univariate Cox Regression Analysis was performed using variables: histologic type (WDNET versus MHT), age at the time of diagnosis (≤65 years old versus >65 years old), gender, race, lymph node involvement, and surgery type (appendectomy/cecectomy versus RHC/or greater). Among all variables, histologic type of MHT, age of >65 years old at the time of diagnosis, African-American race, lymph node involvement and type of surgery (RHC/or greater) were found to be significantly associated with poor survival. In multivariate Cox Regression Analysis, only histologic type, age >65 years old at the time of diagnosis and lymph node involvement remained significant after adjusting for other characteristics (Table 5).

**Table 5:** Univariate and multivariate cox regression analyses for appendiceal WDNETs and appendiceal MHTs for cancer specific survival for appendiceal NETs

Factors	Univariate Analysis		Multivariate Analysis	
	HR (95% CI)	p-value	HR (95% CI)	p-value
Histologic Subtype				
WDNET (n=674)	-Ref-		-Ref-	
MHT (n=1067)	3.60 (2.28-5.68)	<0.001	15.77 (6.81-36.53)	<0.001
Age at the time of diagnosis				
≤65 years old	-Ref-		-Ref-	
>65 years old	3.52 (2.55-4.87)	<0.001	2.18 (1.42-3.36)	<0.001
Sex				
Male	-Ref-		-Ref-	
Female	1.30 (0.97-1.75)	0.08	1.23 (0.83-1.82)	0.30
Race				
Whites	-Ref-		-Ref-	
Blacks	1.62 (1.06-2.47)	0.03	1.09 (0.61-1.96)	0.77
Others	1.14 (0.53-2.43)	0.73	1.14 (0.41-3.17)	0.80
Lymph Node Involvement				
No	-Ref-		-Ref-	
Yes	6.95 (5.05-9.58)	<0.001	11.41 (7.64-17.04)	<0.001
Surgery Type Appendectomy/cecectomy	-Ref-		-Ref-	
RHC/or greater	1.82 (1.24-2.67)	<0.001	1.12 (0.72-1.73)	0.62

Abbreviations: WDNET: well-differentiated neuroendocrine tumor; MHT: mixed histology tumor; HR: hazard ratio; CI: confidence interval; Ref: reference; RHC: right hemicolectomy.

**Figure 3:**
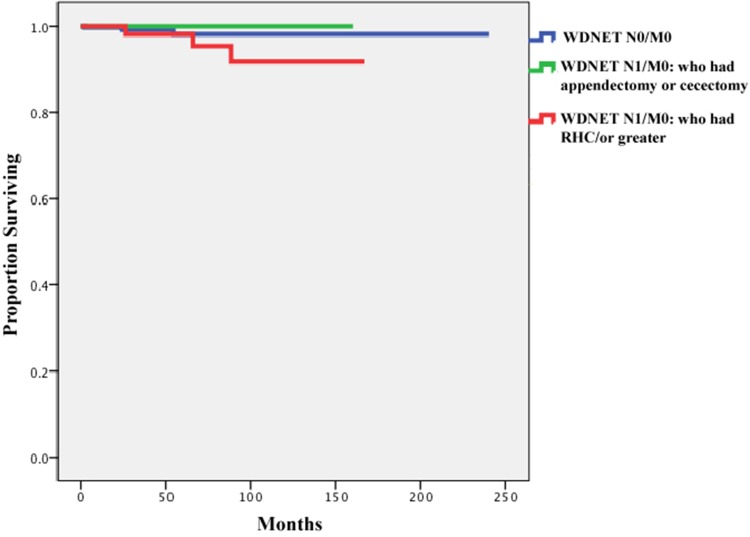
Comparison ofcancer-specificsurvival of patients with well-differentiated neuroendocrine tumors (WDNETs) and no nodal metastases (n=368), those with nodal involvement who underwent appendectomy or cecectomy (n=28), and those with nodal involvement who underwent right hemicolectomy/ or greater (n=90) (date of diagnosis 1988–2012)

**Figure 4:**
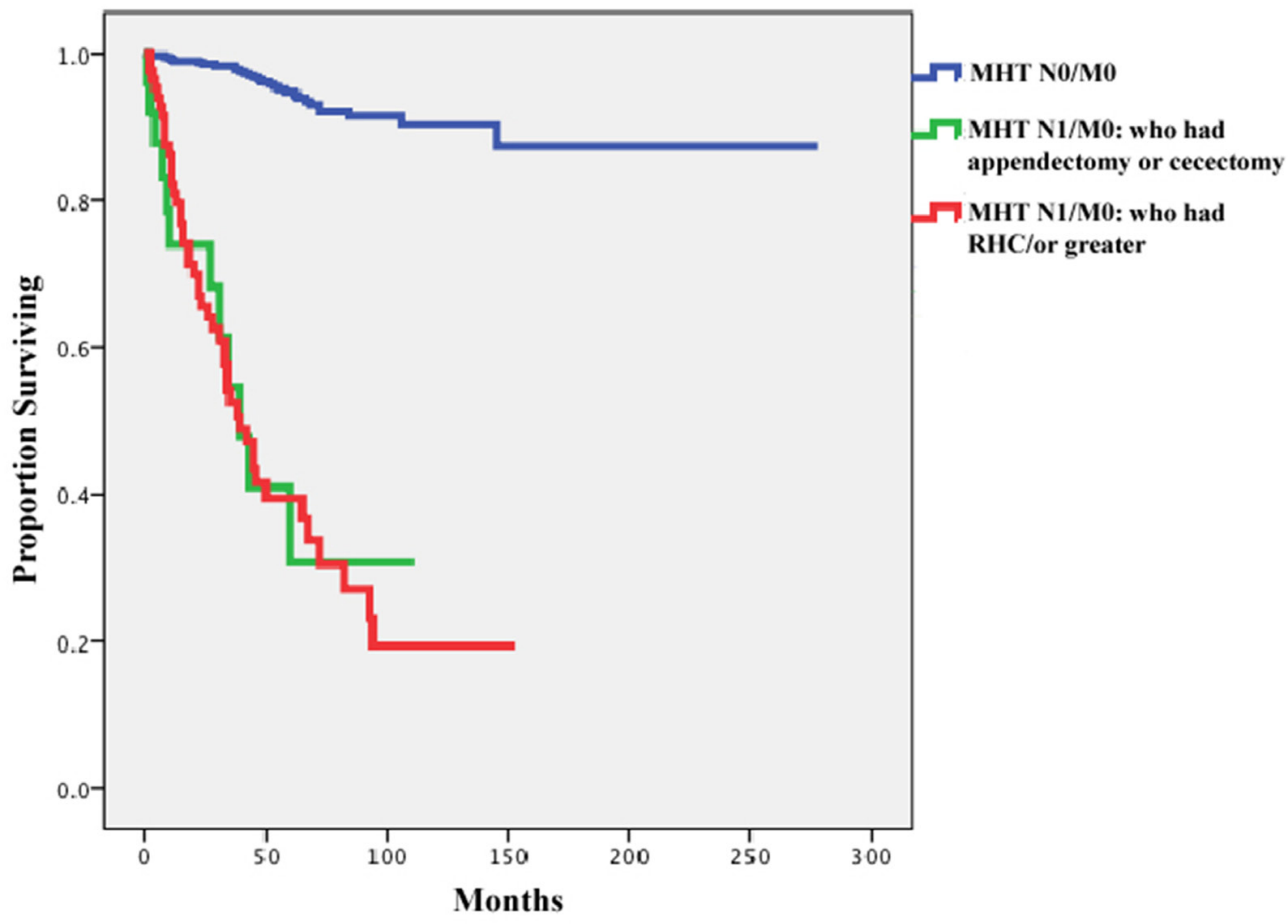
Comparison of cancer specific survival of patients with mixed histology tumor (MHT) and no nodal metastases (n=651), those with nodal involvement who underwent appendectomy or cecectomy (n=25), and those with nodal involvement who underwent right hemicolectomy/ or greater (n=86) (date of diagnosis 1988–2012)

In order to understand the impact of surgical type on patient outcomes in cases with nodal metastases, we compared the survival of patients who had node-positive disease but no distant metastases according to their type of surgery (i.e. appendectomy/cecectomy or RHC) and with those of patients who had no nodal metastases at the time of diagnosis. This comparison was separately performed in each histologic subtype (WDNET and MHT). Patients with PDNEC were excluded from these analyses because of the small available sample size for these rare tumors. In Figures 3 and 4, the cancer-specific survival rates of patients with node-negative disease are compared with those of patients with node-positive disease who underwent appendectomy/cecectomy and those who underwent RHC or greater. The univariate Cox regression analysis showed that the type of surgery did not affect survival in these patients (hazard ratio, 1.00; 95% CI, 0.53–1.89; *p* =0.99). We did not perform this analysis for patients with WDNETs because only 3 died within 10 years reflecting their excellent survival irrespective of lymph node involvement.

## DISCUSSION

To our knowledge, our study is the first to compare the effects of nodal metastases, tumor size, and histologic subtype on the prognosis of patients with appendiceal neuroendocrine neoplasms.

The current guidelines are based on studies by Moertel et al [11, 13]that failed to identify any nodal metastases in carcinoid tumors smaller than 20 mm leading the proposed cut-off of 20mm for RHC. However, we found a higher percentage of regional lymph node involvement (56.7%) in WDNETs of 11–20 mm than have other studies (Moertel et al, 0%; Mullen and Savarese, 47.0%; and Groth et al, 29.9%) [2, 11, 18]. The difference is probably explained by the fact that all the prior studies used the broad definition of “carcinoid tumor” of appendix while, we sub-classified NETs based on the most recent WHO classification. In addition, in contrast to these studies, for the purpose of evaluating the associations between clinicopathologic features and regional lymph node involvement (Tables 2 and 3), we only included cases that had available data for pN0/or pN1 (i.e. cases with only clinical staging data for lymph node involvement were excluded). Finally, we considered patients to have node-negative disease only if they had adequate lymph node dissection (defined as at least 12 lymph nodes examined, extrapolating from colon cancer data given anatomical proximity and similar surgical techniques) and no nodal involvement.

Our results suggest that a tumor size of 10 mm is a more appropriate predictive threshold than 20 mm for predicting lymph node involvement in WDNETs. We also observed a higher rate of nodal metastasis in 11-to 20-mm MHTs than in those smaller than 10 mm (32.9% vs. 10.4%). While patients with WDNETs have a favorable prognosis even when they have nodal metastases, patients with MHTs—despite their lower overall rate of nodal metastases-have dramatically lower survival rates when they have lymph node metastasis. PDNECs are almost always discovered when they have grown larger than 10 mm and harbor a significant risk of both nodal and distant metastases.

Patients with WDNETs had favorable overall outcomes in our study irrespective of lymph node involvement – therefore, extensive resection may not significantly improve survival. This finding agrees with those of other recent studies [2, 18, 19]. Our findings in patients with MHTs and LN involvement suggested that they overall have significantly worse outcomes compared to WDNETs and that also RHC does not improve outcomes. Whether these patients may benefit from post-operative adjuvant chemotherapy remains to be determined. Therefore, we believe that any plan regarding extensive surgery, whether based on tumor size or lymph node involvement, should be personalized according to the potential risks and benefits. For instance, in an elderly patient with multiple comorbidities, a 15-mm WDNET may be reliably managed with simple appendectomy, even if there is regional lymph node involvement.

Our study has several strengths and limitations. Appendiceal NETs are rare and therefore, SEER dataset can serve as an important resource for investigators. Including cases from a national, population-based tumor registry, would lend generalizability of the results. Extensive information collected by SEER program enabled us to analyze outcome after adjusting for age, gender, race, tumor stage, and also to use cancer-specific survival (instead of overall survival). In addition, we grouped SEER histology codes according to the most recent WHO classification of appendiceal tumors and outcomes were analyzed in each histologic subtype (WDNET, and MHT) separately. Our study has some limitations related to its retrospective nature and to the data available in the SEER database. First, since some appendiceal neuroendocrine tumors may be considered benign, not all cases may have been reported to the SEER program. Second, the SEER database does not capture other important prognostic features, such as performance status, comorbidities, recurrence data, lymphovascular invasion, tumor location within the appendix, and adjuvant therapy, which may impact outcome. Finally, we classed appendectomy and cecectomy together, as the SEER data use the same code for both types of surgical resection. Similarly, RHC and any type of more extensive RHC were coded identically in the database; therefore, both were categorized as “RHC” in our analysis.

In summary, our population-based study suggests that although WDNETs have a much higher rate of nodal metastasis in tumors sized 11–20 mm than previously shown. The generally favorable outcomes for patients with WDNETs do not seem to be further improved by RHC. Therefore, we recommend that appendectomy be considered an adequate treatment option for select WDNET cases even in the presence of nodal metastases. Conversely, in patients with MHTs, nodal involvement significantly reduces survival rates, which were not improved by RHC. Future studies should evaluate the role of adjuvant chemotherapy in this subgroup.

## MATERIALS AND METHODS

Data were collected from the SEER public-use database of the National Cancer Institute (November 2014 submission), which includes data from 18 cancer registries [20]. The study did not require Institutional Review Board approval according to our institutional guidelines.

### Histology codes

Histology codes were obtained from the third edition of the International Classification of Diseases for Oncology and its supplements and were grouped according to the 2010 WHO classification of appendiceal tumors [4]. WDNETs included appendiceal tumors with the following SEER histology/behavior codes: 8246/3 “neuroendocrine carcinoma, not otherwise specified (NOS)” (grades 1 and 2 only); 8240/3 “neuroendocrine tumor, low grade;” 8241/3 “enterochromaffin cell carcinoid;” 8242/3 “enterochromaffin-like cell tumor, malignant;” and 8249/3 “neuroendocrine tumor, grade 2.” PDNECs were identified with the SEER histology/behavior codes 8012/3 “large cell carcinoma, NOS;” 8013/3 “large cell neuroendocrine carcinoma;” 8014/3 “large cell carcinoma with rhabdoid phenotype;” 8041/2 and 8041/3 “small cell carcinoma, NOS;” 8042/3 “oat cell carcinoma;” 8043/3 “small cell carcinoma, fusiform cell;” 8044/3 “small cell carcinoma, intermediate cell;” 8045/3 “combined small cell carcinoma;” and 8246/3 “neuroendocrine carcinoma, NOS” (grades 3 and 4 only). MANEC is defined as a tumor with both neuroendocrine and epithelial components when each component constitutes greater than 30% of neoplastic tissue. Therefore, they are thought to warrant a treatment approach similar to that of colon tumors [4–6]. Since the ratio of the histologic components has not been recorded in SEER database, we classified tumors with both neuroendocrine and epithelial components as “Mixed histology tumors (MHTs)”. MHT was identified with histology/behavior codes 8243/3 “goblet cell carcinoid;” 8244/3 “mixed adenoneuroendocrine carcinoma;” and 8245/3 “adenocarcinoid tumor.”

### Evaluation of associations between clinicopathologic features (including tumor size) with regional lymph node involvement in each histologic subtype

For all 3 histologic subtypes, we used the seventh edition of the American Joint Committee on Cancer (AJCC) Staging Handbook for TNM classification and staging [21]. In this edition, the guideline for TNM classification and staging of WDNETs is substantially different from that for appendiceal carcinomas [1]. In addition to tumor extension, tumor size is now considered major factor in determining the T classification of WDNETs.

In SEER data, collaborative staging (CS) variables, which have been recorded since 2004, can be reliably used to render TNM classifications. For cases diagnosed before 2004, the “extent of disease” variable was used to code tumor extension; unfortunately, because this code lacks specificity, it cannot be reliably rendered to T stage using the current staging guidelines. Therefore, in order to accurately represent the relationship between nodal status and TNM staging and tumor size, we decided to include only patients diagnosed after 2004 for whom CS variables, including “CS-tumor size” and “CS-tumor extension,” were available.

Using the variable “CS-tumor size,” we classified tumor size into 3 categorical variables: ≤10 mm, 11–20 mm, and >20 mm. The presence of regional lymph node involvement was recorded as binary variable. In this analysis, we only included patients who had pathologic evidence of lymph node involvement. According to AJCC 7th Ed - Appendix chapter, “Histological examination of a regional lymphadenectomy specimen ordinarily includes 12 or more lymph nodes”. Therefore, patients were classified as having node-negative disease only if adequate lymphadenectomy data (defined as 12 or more reported regional lymph nodes) were available and all examined lymph nodes were negative. Node-positive disease was defined as any pathologic lymph node involvement, regardless of the number of examined lymph nodes.

### Evaluation of associations between clinicopathologic characteristics and type of surgery with overall survival

Patients diagnosed from 1988 to 2012 who had available demographic and staging data were included in survival analyses. In the survival analyses, the staging basis for lymph node involvement was clinical (and not pathologic). Patients diagnosed before 1988 were excluded because data regarding lymph node involvement were not uniformly recorded in the SEER database before this point. Patients were excluded from the survival analyses if they had distant metastasis; if they had no follow-up information; if the reporting source was a hospice, nursing home, autopsy, or death certificate; if they had more than 1 primary tumor or more than 1 malignancy during their lifetime; or if the cause of death was unrelated to appendiceal cancer. Surgical treatment was categorized as no surgery, local surgery, appendectomy or cecectomy, and RHC or extended hemicolectomy.

### Statistical analyses

Patient demographic and clinicopathologic characteristics were obtained from SEER database and categorical variables were compared using the χ^2^ test. One-way analysis of variance (ANOVA) was used to compare the means of continuous variable (i.e. age) between groups. Survival durations were compiled using the Kaplan-Meier method and compared using the log-rank test. Univariate and multivariate Cox regression analyses were performed to determine the association of various factors with survival. All *p* values were 2-sided, and statistical significance was set at *p* <0.05. Analyses were performed using SPSS software for Windows version 16.0 (SPSS Inc., Chicago, IL).

## SUPPLEMENTARY MATERIALS TABLE



## Abbreviations

NET: neuroendocrine tumor
WDNET: well-differentiated neuroendocrine tumor
PDNEC: poorly differentiated neuroendocrine carcinoma
MANEC: mixed adenoneuroendocrine carcinoma
MHT: mixed histology tumor
LN: lymph node
NOS: not otherwise specified
HR: hazard ratio
CI: confidence interval
Ref: reference
RHC: right hemicolectomy
AJCC: American Joint Committee on Cancer
SEER: Surveillance, Epidemiology, and End Results
OS: overall survival
ANOVA: analysis of variance
CS: collaborative staging
