# Supplementary material for: Regional lymph node involvement and outcomes in appendiceal neuroendocrine tumors: a SEER database analysis
Source: Oncotarget. 2017 Aug 19;8(59):99541–51. doi: 10.18632/oncotarget.20362 (PMC5725113; doi:10.18632/oncotarget.20362)
Supplement: Supplementary file 1 [file oncotarget-08-99541-s001.pdf]

Regional lymph node involvement and outcomes in appendiceal neuroendocrine tumors: a SEER database analysis

SUPPLEMENTARY MATERIALS

Supplementary Table 1: Frequency of appendiceal neuroendocrine tumor histologies in surveillance, epidemiology, and end results data (date of diagnosis 1988–2012)

Tumor type	Histology Code	Definition	N
WDNET	8240/3	Neuroendocrine tumor, low grade	643
(n=860)	8241/3	Enterochromaffin cell carcinoid	14
	8246/3	Neuroendocrine carcinoma, NOS (Grade 1 and 2)	188
	8249/3	Neuroendocrine tumor, grade 2	15
PDNEC	8013/3	Large cell neuroendocrine carcinoma	*
(n=26)	8041/3	Small cell carcinoma, NOS	*
	8246/3	Neuroendocrine carcinoma, NOS (Grade 3 and 4)	*
MHT	8243/3	Goblet cell carcinoid	911
(n=1659)	8244/3	Mixed adenoneuroendocrine carcinoma	247
	8245/3	Adenocarcinoid tumor	501

Abbreviations: WDNET: well-differentiated neuroendocrine tumor; PDNEC: poorly differentiated neuroendocrine carcinoma; MHT: mixed histology tumor; NOS: not otherwise specified * In compliance with SEER Program Data Use-Agreement, the small numbers of cases with PDNEC are not reported.
